# Filter paper-based spin column method for cost-efficient DNA or RNA purification

**DOI:** 10.1371/journal.pone.0203011

**Published:** 2018-12-07

**Authors:** Rui Shi, Ramsey S. Lewis, Dilip R. Panthee

**Affiliations:** 1 Department of Horticultural Science, North Carolina State University, Mountain Horticultural Crops Research & Extension Center, Mills River, NC, United States of America; 2 Department of Crop and Soil Science, North Carolina State University, Raleigh, NC, United States of America; Helsingin Yliopisto, FINLAND

## Abstract

We describe herein a method of recharging used commercial spin columns or assembling homemade spin columns using filter paper as binding material for cost-effective, low throughput nucleic acid purification. The efficiency of filter paper-based spin columns was evaluated for purification of nucleic acids from various sources. Following protocols of commercial kits, we found filter paper to be a useful binding material for purification of nucleic acids, including plant genomic DNA, plant total RNA, PCR products, and DNA from agarose gels. However, filter paper has a weak binding affinity to plasmid DNA in tested miniprep protocols. Protocols for the use of filter paper recharged spin columns or homemade spin columns for low throughput purification of plant genomic DNA and total RNA with unused commercial kit buffers or less expensive homemade buffers are presented.

## Introduction

Nucleic acid purification is the starting point for many applications in molecular biology [1, 2]. Classical methodologies for purification of nucleic acids are based upon organic extraction followed by ethanol-based precipitation. However, these methods are time-consuming and require the use of toxic solvents, such as chloroform and phenol, which can be harmful to the user and the environment [1, 2].

Commercial nucleic acid purification kits usually involve solid-phase approaches whereby nucleic acids extracted in solution are absorbed by a solid-phase binding material under chaotropic conditions, followed by washing of non-nucleic acids remaining on the binding material using appropriate buffer solutions, and elution of purified nucleic acids from the binding material using low salt solutions [1, 3, 4]. Silica-based materials in matrix form (glass fiber filters or silica membranes) are the widely adopted nucleic acid binding material, and guanidine-based buffers are the commonly adopted for providing a chaotropic condition for nucleic acid binding [1]. Methods involving the use of plant-based cellulose material have also been described for nucleic acid purification. Su and Comeau [5] found cellulose fibers to be of value for purification of nucleic acids, with MeganCel paramagnetic cellulose particles (Promega, Madison, WI) being an example of a commercial product [6]. Additionally, Diethylaminomethyl (DEAE)-modified cellulose can serve as a DNA binding material in column-based nucleic acid purification [7]. Commercial kits using a solid phase approach provide for fast purification of high-quality nucleic acids and have been used extensively by molecular biology laboratories. Such kits are expensive, however.

Commercial kits usually contain spin columns or multiple well spin plates assembled with solid-phase nucleic acid binding material for easy binding, washing, and elution of nucleic acids in the purification process. Commercial spin columns or plates are designed for one-time use, but their cost is even higher total cost of many classic purification methods. Many labs attempt to reduce operating expenses by reusing spin columns or plates after decontamination and regeneration using commercial kits [8, 9] or treatment using in-house protocols [10]. However, commercial regeneration products, such as the maxXbond nucleic acid purification system (AppliChem GmbH) require additional cost to purchase, and less expensive in-house protocols require HCl and careful handling [10]. Preparation of homemade spin column or plates may be an efficient, cost-effective alternative approach. For example, Borodian et al [11] describe homemade spin columns for plasmid DNA purification through insertion of silica-based glass fiber filters into a 0.5 ml tube with holes punched at the tube bottom.

We noted in a previous report that filter paper made from cellulose fiber can be a superior replacement for silica-based material in preparing 96-well spin plates for high throughput purification of plant genomic DNA from CTAB/NaCl extraction [12]. Another recent report indicated that filter paper tips can be used for quick purification of nucleic acids from crude extracts for PCR based analyses [13]. Cellulose-based filter paper is inexpensive and readily available in laboratories. Therefore filter paper should have widespread utility for solid-phase nucleic acid purification. Here, we describe the use of filter paper in recharging of used commercial spin columns or creation of homemade spin column for low throughput nucleic acid purification.

## Materials and methods

### Plant materials, plasmid DNA and PCR products

Tomato (*Solanum lycopersicum* L.) leaf samples were collected from field or greenhouse-grown plants at Mountain Horticultural Crops Research & Extension Center of North Carolina State University at Mills River, NC. USA. Tobacco (*Nicotiana tabacum* L.*)* leaf samples were collected from laboratory or growth chamber experiments being carried out by the Department of Crop and Soil Science at North Carolina State University, Raleigh, NC. USA. For DNA purification, collected fresh samples were used immediately or stored at -20°C before use. Usually, about 50 to 100 mg of leaf tissue was collected for each sample and placed into 2 ml screw cap tubes for grinding using a mechanical homogenizer, or into 1.5 ml Eppendorf tubes for grinding using plastic pellet pestles. Leaf samples for RNA purification were immediately frozen in liquid nitrogen after collection and ground into a fine powder in liquid nitrogen. 50 to 100 mg ground samples were transferred into a tube and used immediately or stored at -80°C prior to use.

pUC19 and pBI121 plasmids were purchased from Invitrogen (Carlsbad, CA). The GUS gene fragment was amplified from the pBI121 plasmid DNA or DNA purified from transgenic tobacco by PCR using the following primer set:

Forward, 5’- TGACCTCGAGGTCGACGATATCGTCGTCATGAAGATGCGGAC- 3’, and

reverse, 5’- CTAGACTAGTCCCGGGGGTACCATCCACGCCGTATTCGGTG-3’.

### Filter paper-based spin column preparation

**Fig 1:**
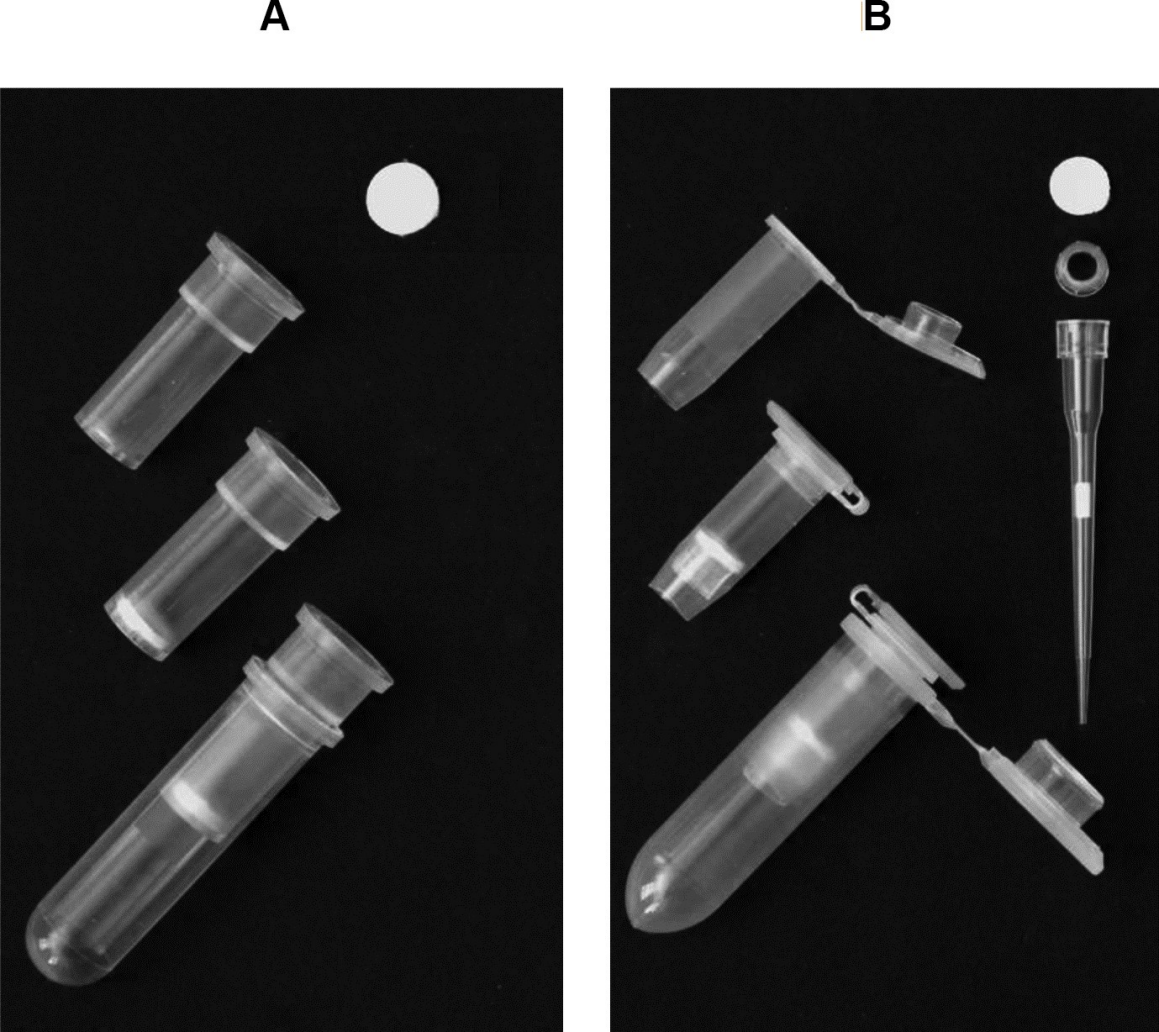
Recharging commercial spin columns and homemade spin columns using filter paper. (A) Recharged commercial spin columns with a flat bottom and net structure to support filter paper discs. (B) Homemade filter paper-based spin columns prepared based on 0.5 ml tubes fitted with a head part of a 10 μl pipette tip (ART 10 REACH model, Molecular BioProducts) to support filter paper discs.

Recharging of used commercial spin columns was initiated by separating plastic parts and treating them with a 1:10 dilution of bleach (~0.6% sodium hypochlorite after dilution) for at least 10 min, followed by triple rinsing with sterilized water and air-drying. The preferred spin column format has a flat bottom with a net-like structure to support binding matrix (Fig 1A). To recharge spin columns with this format, we loaded one or two layers of filter paper discs into the column by pushing the discs into the bottom using the end of a 200 μl pipette tip (Fig 1A). Filter paper discs were punched from sheets of Whatman qualitative filter paper, Grade 3 (GE Healthcare Life Science, UK) or equivalent filter paper using a 5/16-inch (~8mm) paper punch.

Creation of homemade spin columns was based on standard 0.5 ml Eppendorf tubes. The lower part of centrifuge tubes was cut off using a razor blade as shown in Fig 1B, and the head or collar part (proximal body region that receives pipette mandrel) of 10 μl pipette tips (e.g. ART 10 REACH model, Molecular BioProducts/Thermo Scientific) was inserted into the tube to serve as supporting ring (Fig 1B). One or two layers of filter paper discs were then loaded onto the supporting ring in the tube and pushed tightly using the end of 10 μl tip (Fig 1B). Filter paper discs for such homemade spin column were punched from filter paper sheet (Grade 3, GE Healthcare Life) using a standard 1/4-inch (6.35 mm) paper punch.

The assembled filter paper-based spin columns were placed into cleaned 2 ml collection tubes from a commercial kit (Fig 1A), or 2 ml new centrifuge tubes (Fig 1B), and autoclaved. Autoclaved spin columns along with collection tubes were air dried and stored in a dry place prior to use.

### Purification of plant genomic DNA

Plant genomic DNAs were purified using filter paper-based spin columns following the modified protocol of the Qiagen DNeasy Plant mini kit (Qiagen DNeasy plant handbook, March 2018 version) or an in-house protocol using homemade buffers described by Lemke et al [9].

To lyse plant material, 400 μl of Qiagen kit AP1 buffer or homemade lysis buffer (0.5% SDS, 8% PVP-10, 250 mM NaCl, 25 mM Na-DETA, 200 mM Tris-HCl pH7.5) was added with 4 μl RNase solution (100 mg/ml, Qiagen) into a 2 ml screw cap tube containing 50 to 100 mg frozen or fresh plant material. Two tungsten carbide beads (Qiagen) or similar beads were added into the tube. Samples were homogenized using a FastPrep FP120 cell homogenizer (Savant Instruments Inc, Holbrook, NY) according to manufacturer’s instructions. Alternatively, 50 to 100 mg frozen or fresh samples were ground in a 1.5 ml Eppendorf tube with lysis buffer and RNase using disposable plastic pellet pestles (USA Scientific, Ocala, FL).

Tubes with lysis mixtures were incubated at 65°C for 10 min and inverted 2 to 3 times during incubation. After mixing with 130 μl P3 buffer (Qiagen) or 130 μl homemade precipitation buffer (5M KAc, pH 6.5), lysis mixtures were incubated on ice for at least 5 mins. The lysate was cleared through centrifugation at top speed (≥ 16,000 × g) for 10 mins. In cases where the lysate is not clear, it can be transferred to a spin column (with one layer of filter paper) placed in a new 2 ml tube used as a collection tube and centrifuged at 6,000 × g for 1 min followed by a second round of centrifugation at top speed for 1 min to obtain clear lysate.

To bind DNA to filter paper discs, clear lysate was transferred into a new tube and mixed with 1.5 × volume of AW1 buffer (Qiagen) or homemade binding buffer (2M guanidine hydrochloride, 75% ethanol) by pipetting or inversion, then transferred to a new filter paper-based spin placed in a new 2-ml collection tube. These tubes were centrifuged at 6000 × g for 1 min, and flow through discarded. The remaining lysate was loaded into a spin column for another round of centrifugation so that all the lysate mixture was passed through the filter paper discs.

To wash the filter paper discs in the spin column, 500 μl AW2 buffer (Qiagen) or homemade washing buffer I (10 mM NaCl, 10 mM Tris-HCl pH 6.5, 80% ethanol) was added to the column and centrifugation at 6000 × g for 1 min was performed. Flow through was discarded and the spin column was placed into the same collection tube. A second wash was done with 500 μl AW2 buffer (Qiagen) or homemade washing buffer II (95% ethanol) and spun at 6000 g for 1 min. Spin column was centrifuged at top speed (≥16,000 × g) for at least 2 mins.

Spin columns were inserted into new 1.5 ml tubes, and air dried for 10 mins. 100 μl AE buffer (Qiagen kit) or 10 mM Tris-HCl (pH8.5) was added to the filter paper in the spin column and incubated at room temperature for 5 mins. DNA elution was collected into collection tube through centrifugation at 6000 × g for 1 min.

### Purification of plant RNA

Plant total RNAs were purified using filter paper-based spin columns following the protocol of the Qiagen RNeasy Plant mini kit (RNeasy Mini Handbook. Fourth edition, June 2012) or a protocol using homemade buffer presented by Yaffe et al [14].

Up to 100 mg of ground frozen sample collected in 1.5 or 2 ml tube was suspended in 450 μl RTL or RLC buffer (Qiagen kit) with 1% (v/v) *β*-mercaptoethanol or homemade lysis buffer (8 M guanidine hydrochloride, 20 mM MES and 20 mM EDTA) with 1% (v/v) *β*-mercaptoethanol.

The tube with lysis mixture was incubated at 55°C for 1 to 3 min and mixed by inverting 2 to 3 times during incubation, and then centrifuged in a microcentrifuge at top speed (≥ 16,000 × g) for 10 mins to clear the lysate. Optionally, the lysate was transferred into a spin column (one layer of filter paper) and centrifuged at 8,000 × g for 1 min followed by another round of centrifugation at top speed for 1 min.

Entire clear lysate was transferred to a new 1.5 ml tube and mixed with a half volume of ethanol by pipetting or inversion. Lysate-ethanol mixture was transferred into a new spin column assembled with two layers of filter paper disc and centrifuged at 8000 × g for 1 min. After discarding the flow through, remaining lysate mixture was transferred to spin column and centrifuged at 8000 × g for 1 min.

To wash the column, 700 μl of RW1 buffer (Qiagen) or homemade washing buffer I (3 M Na-acetate pH 5.2) was added to the spin column, followed by centrifugation at 6000 × g for 1 min. Flow through was discarded, and 500 μl RPE buffer (Qiagen) or homemade washing buffer II (70% ethanol) was added to the column and centrifuged at 8000 × g for 1 min. Spin columns were transferred to new collection tubes and centrifuged at top speed for at least 2 mins to eliminate remaining ethanol. Spin columns were inserted into a new 1.5 ml collection tube, allowed to air dry, and 50 μl RNase free water was added. Tubes were maintained at room temperature for 1 min, and RNA was eluted into collection tubes by spinning at 8000 × g for 1 min.

### Evaluation of purified nucleic acids

DNA yield and quality were evaluated using 1.0 to 1.5% agarose gel electrophoresis stained with Ethidium Bromide (EtBr). RNA integrity and quality were evaluated using 1% MOPs formaldehyde denaturing agarose gel electrophoresis with EtBr in RNA loading buffer [15]. EtBr stained DNA or RNA fluorescence was visualized and recorded on a Bio-Rad Gel Doc XR+ gel image system (Bio-Rad Laboratories, Hercules, CA), or a FOTODYNE system (FOTODYNE Incorporated, Hartland, WI). DNA and RNA quality and quantity were also evaluated using a Nanodrop 2000 UV spectrometer (Fisher Scientific, Waltham, MA).

Quantitative PCR (qPCR) and quantitative reverse transcription PCR (qRT-PCR) were used to determine whether the purified DNA or RNA contained contamination which could interfere with the efficiency of PCR. Briefly, 10 μl SYBR Green-based qPCR mix was prepared using Luna Universal Probe qPCR Master Mix (New England Biolabs, Ipswich, MA, USA) with 0.5 μM primer set of Tubulin gene for tobacco [16]. For DNA evaluation, we added 20 ng genomic DNA purified by different methods. For RNA evaluation, cDNAs were reverse transcribed from 200 ng of purified RNA in a 10 μl reverse transcription reaction prepared using iScript cDNA Synthesis Kit (Bio-Rad), and cDNA of 2.5 ng RNA was added to the qRT-PCR reaction. Both qPCR and qRT-PCR were run on CFX96 Real-Time PCR System following a standard two-step PCR program as suggested by Luna Universal Probe qPCR Master Mix manual. Amplification of different input templates were evaluated using CFX Maestro Software v1.1 (Bio-Rad) based on quantification cycle (Cq) value, the calculated fractional cycle number at which the target DNA amplicon associated fluorescent accumulated to an arbitrary threshold [17].

## Results

### Recharged spin columns and homemade spin columns using filter paper

Our efforts to prepare a filter paper-based spin column were initiated by recharging used commercial spin columns with filter paper disc(s). Recharged spin columns can be easily prepared based on a preferred commercial spin column with a flat bottom and a net structure supporting the binding material, such as those Wizard SV minicolumns from Promega (Madison, WI) (Fig 1A, S1 Fig). Alternatively, spin columns with a conical (V-shape) bottom and a drip opening, such as a miniprep column from Qiagen (S1 Fig), and a recent version adopted in NEB Monarch plasmid miniprep kit, can be recharged by reloading filter paper discs with a diameter of 5/16 inch (~8 mm) (S1 Fig). NEB version columns may also be loaded with filter paper discs of 3/16 inch (~5mm) (S1 Fig). Because filter paper is relatively strong and holds its shape better than a soft silica-based glass fiber filter, fixing rings (O-ring) and support plastic frits used in many commercial spin columns are not required. Exclusion of these items helps to simplify the recharging process and avoid leftover solution on the fixing ring during purification.

In an earlier report, Borodina et al. [11] prepared a spin column by adding glass filter paper to the bottom of a 0.5 ml tube with several small holes punched at the bottom. However, this method does not work for filter paper, since filter paper discs are hard to push down to the bottom of the tube evenly as soft glass fiber filter. If we push filter paper too hard to fill the tube bottom, it might be too tight to block flow through. Otherwise, it leaves space and results in sample leakage.

We found that we can create a simple homemade spin column by loading filter paper disc(s) on the head part of a 10 μl pipette tip as a supporting ring in the tube (Fig 1B). The preferred 10 μl pipette tip is ART 10 (REACH) model (Fig 1B) or 10 μl XL TipOne RPT tip (Cat 1160–3700, USA Scientific. S1 Fig). The head part of these tips has projected fins and can leave space between the supporting ring and the inner wall of the tube. Such space allows the solution to flow through the filter paper without leftover (Fig 1B, S1 Fig). Other tips such as 10 μl ART 20E model are not optimal as the heads of these tips are too large to be inserted at the desired position within a 0.5 ml tube.

Both recharged, or homemade filter paper-based spin column can be easily disassembled, cleaned, and recharged with filter paper discs for repeated use.

### Evaluation of filter paper in the purification of nucleic acids from various sources

Our initial experiments suggested that filter paper may function in commercial kits for nucleic acid purification. To investigate this possibility, we reassembled spin columns of Qiagen kits by replacing the original silica-based membranes with filter paper discs, then using the reassembled spin columns for purification of nucleic acids from various sources using respective Qiagen kits in our hands, including the DNeasy plant mini kit for plant genomic DNA, the RNeasy plant mini kit for plant RNA, the QIAquick PCR purification kit for PCR products, the QIAquick gel extraction kit for DNA extraction from agarose gels, and the QIAprep spin miniprep kit for plasmid DNA. For comparison of effectiveness, we also included original spin columns and reassembled spin column using glass fiber filters (Whatman glass microfiber filters, Grade GF/F, GE), a silica-based material usually adopted for recharging or preparing homemade spin column [9, 11].

**Fig 2:**
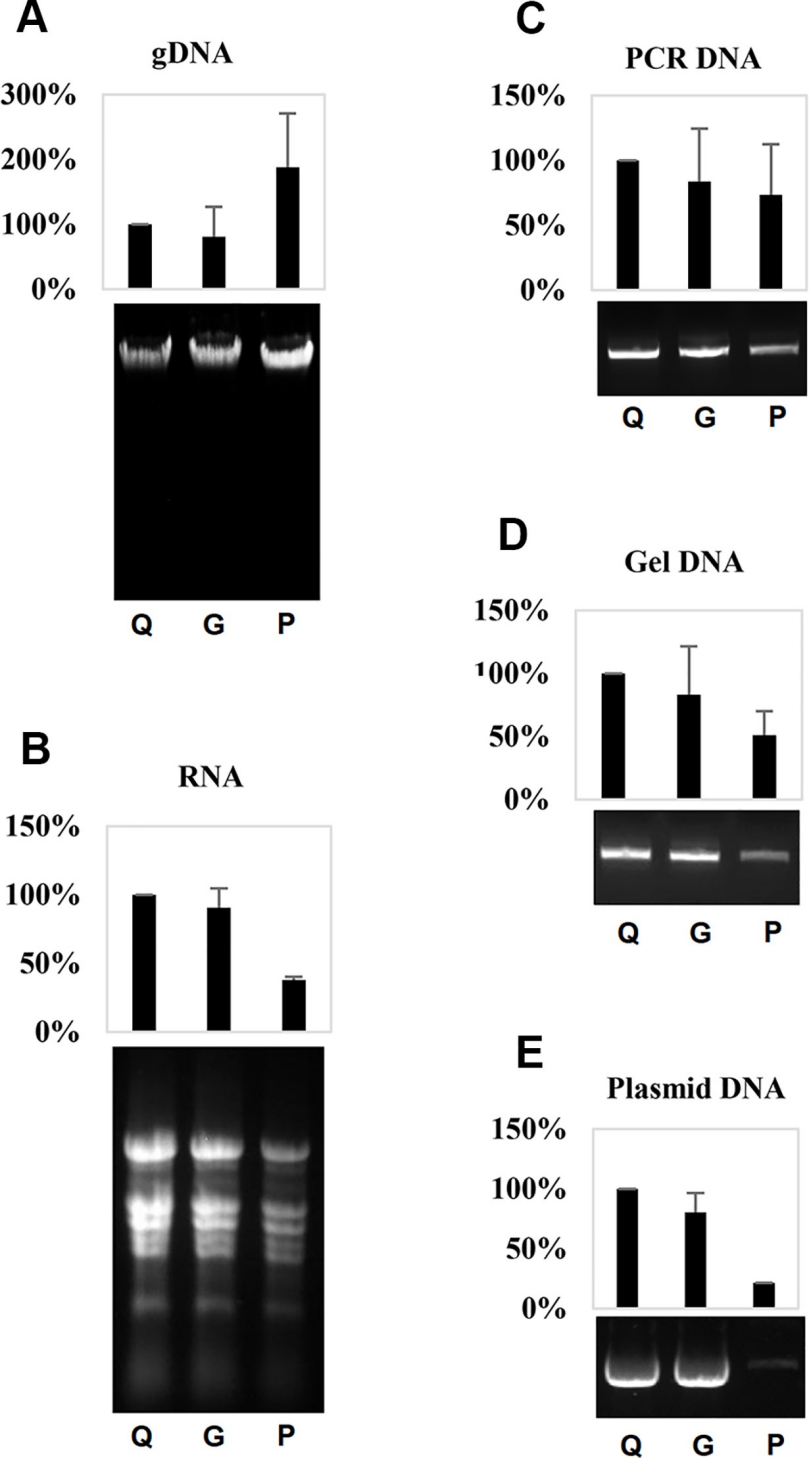
The efficiency of filter paper for purification of nucleic acids from various sources using respective Qiagen kits. (A) Tomato genomic DNAs purified using Qiagen DNeasy plant mini kit. (B) Tomato total RNAs purified using Qiagen RNeasy plant mini kit. (C) PCR products of a GUS fragment purified using Qiagen QIAquick PCR purification kit. (D) PCR products of GUS fragment recovered from an agarose gel using a Qiagen QIAquick gel extraction kit. (E) *pUC*-19 plasmid DNAs purified using a Qiagen QIAprep spin miniprep kit. For each panel, from left to right are (Q) nucleic acid purified in experiments using original Qiagen spin column, (G) reassembled spin column using two layers of Whatman glass microfiber filters (Grade GF/F), and (P) reassembled spin column using two layers of Whatman qualitative filter paper, (Grade 3) respectively. Upper panel is quantification data based on three experimental replicates normalized according to performance of the Qiagen kit; lower panel is an image of agarose gel electrophoresis for the same volume of purified nucleic acids.

To our surprise, the filter paper based-spin columns exhibited superior performance for purification of tomato genomic DNA and yielded higher final DNA quantities than the original Qiagen spin columns or the glass fiber-reassembled spin columns (Fig 2A). Filter paper-based spin columns were also able to purify plant total RNA (Fig 2B), DNA from PCR (Fig 2C), and to recover DNA from agarose gels (Fig 2D), although the yields of these nucleic acids were relatively lower compared to yields from the silica-based spin columns (Fig 2). Apart from these findings, we noticed that filter paper reassembled spin columns do not work well for purification of plasmid DNA using the QIAprep spin miniprep kit protocol, as indicated by weak plasmid DNA bands detected in agarose gel electrophoresis and lower concentrations measured using a UV spectrum meter (Fig 2E).

Based upon these results, we conclude that filter paper works well in the purification of long and linear double-stranded nucleic acids, such as plant genomic DNA following the protocol for silica-based binding material. However, filter paper-based approaches were substantially less effective for supercoiled plasmid DNAs. Nevertheless, it was found that filter paper can serve as an alternative binding material to replace silica-based materials for purification of nucleic acids from many sources, even following the protocols optimized for silica-based materials.

### Purification of plant DNA or RNA using filter paper-based spin columns with commercial kit buffer or homemade buffer

We expected that filter paper-based spin columns could be used as a substitute for commercial spin columns using the remnant buffer of commercial kit to save financial resources. Also, filter paper-based spin columns may work with homemade buffers to further reduce expenses. For instance, several in-house protocols have been developed for silica-based purification of plant DNA [9, 18–20] or RNA [14, 21].

**Fig 3:**
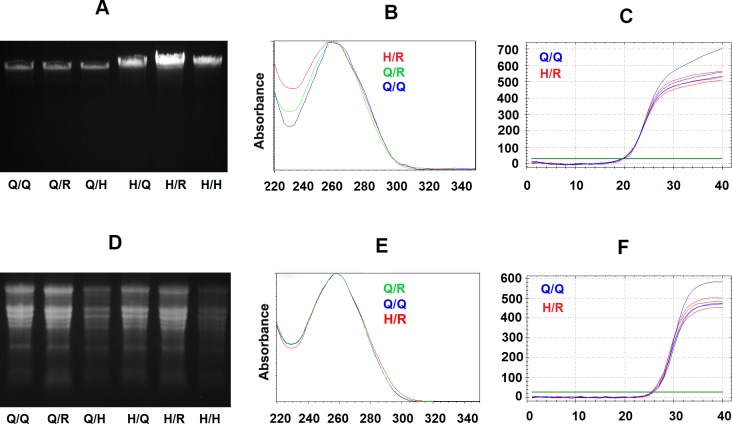
Evaluation of purification of tobacco genomic DNA and total RNA using filter paper-based spin columns with respective Qiagen kit buffers and homemade buffers. (A) Agarose gel electrophoresis for 2.5 μl tobacco genomic DNAs elution from purification experiments using Qiagen DNeasy plant mini kit buffers with Qiagen original spin column (Lane Q/Q), filter paper recharged used spin column (Lane Q/R) and filter paper-based homemade spin column (Lane Q/H*), followed by tobacco genomic DNAs purified using homemade buffer with Qiagen original spin column (Lane H/Q), filter paper recharged used spin column (Lane H/R) and filter paper-based homemade spin column (Lane H/H*). (B) UV spectrum curve of tobacco DNAs purified using Qiagen kit (Q/Q, black curve), filter paper recharged spin columns with Qiagen kit buffers (Q/R, blue curve) or homemade buffers (H/R, red curve) from the same amount leaf tissue. Y-axis is UV absorbance, and X-axis is wavelength (nM). (C) Amplification plots for three duplicated qPCR reactions contain 20 ng DNA purified using Qiagen kit (Q/Q, Blue curves) or DNA purified from filter paper recharged spin column with homemade buffer (H/R, Red curves) respectively. The x-axis is PCR cycle numbers, Y-axis is the level of SYBR fluorescence, and the green line is an arbitrary threshold to determine the Cq value (the fractional cycle number at which amplification curve meet threshold level). (D) MOPS-formaldehyde denaturing agarose gel electrophoresis separated 5 μl RNA purified using Qiagen RNeasy plant mini kit buffers with a Qiagen original spin column (Lane Q/Q), filter paper recharged used spin column (Lane Q/R) and homemade filter paper-based spin column (Lane Q/H*), followed total tobacco RNAs purified by using homemade buffer with Qiagen original spin column (Lane H/Q), filter paper recharged used spin column (Lane H/R) and filter paper-based homemade spin column (Lane H/H*). (E) UV spectrum of tobacco total RNA purified using Qiagen kit (Q/Q, black curve), filter paper recharged spin column with Qiagen RNeasy plant mini kit buffers (Q/R, blue curve) or homemade buffers (H/R, red curve). Y-axis is UV absorbance, and the X-axis is wavelength. (F) Amplification plots of three duplicated qRT-PCR reactions for 2.5 ng RNA purified using Qiagen kit (Q/Q, Blue curves) or RNA purified using filter paper recharged spin column with homemade buffer (H/R, Red curves) respectively. Note: * The starting material amount is 100 mg tobacco leaf tissue for experiments using a Qiagen spin column or filter paper recharged spin column, and half amount of plant sample (50 mg) used for homemade spin column purification. All DNAs or RNAs were eluted using 100 ul elution solution.

As demonstrated in Fig 3, we were able to successfully purify tobacco genomic DNA using filter paper-based recharged or homemade spin column following protocols using Qiagen kit buffers or an in-house protocol modified from Lemke et al [9] using homemade buffers. In Fig 3A, agarose gel electrophoresis clearly indicate the EtBr stain bands of genomic DNA purified from the tobacco leaf using different methods to be similar high molecular weight bands, suggesting similar integrity. Interestingly, the DNA band signals also indicated that higher yields of genomic DNAs could be obtained using filter paper-based spin columns or using homemade buffers compared to the Qiagen kit (Fig 3A), which is similar as results revealed using UV spectrum analysis (Table 1).

**Table 1:** Characterization of purified DNAs and RNAs from tobacco leaf.

Method		DNA			RNA		
Buffer	Column	Yield (μg) per 100 mg leaf	260/280nm absorbance ratio	260/230nm absorbance ratio	Yield (μg) per 100 mg leaf	260/280nm absorbance ratio	260/230nm absorbance ratio
Qiagen	Qiagen	1.67 ± 0.70	2.23 ± 0.49	2.93 ± 4.03	14.4 ± 1.2	2.15 ± 0.01	2.29 ± 0.07
	Recharged	4.52 ± 0.32	1.98 ± 0.08	1.21 ± 0.10	17.2 ± 4.1	2.13 ± 0.01	1.78 ± 0.35
	Homemade	2.85 ± 1.14	1.98 ± 0.18	1.23 ± 0.10	16.0 ± 4.8	2.12 ± 0.02	1.81 ± 0.58
Homemade	Qiagen	5.65 ± 0.72	1.81 ± 0.05	5.03 ± 3.12	11.1 ± 6.3	2.13 ± 0.01	2.37 ± 0.05
	Recharged	7.06 ± 0.81	1.73 ± 0.04	1.53 ± 0.38	15.7 ± 8.8	2.06 ± 0.05	2.32 ± 0.15
	Homemade	5.53 ± 0.88	1.61 ± 0.11	1.37 ± 0.34	18.0 ± 6.4	2.00 ± 0.03	2.46 ± 0.04

UV spectrum analysis also indicated stranded DNA absorbance curve with the peaks at 260 nm for tobacco leaf DNA purified using a filter paper-based spin column with either a Qiagen kit or homemade buffer (Fig 3B), with the exception of relatively low 260/230 ratios (1.2 to 1.5) for DNAs purified from the filter paper-based spin column (Table 1). Low 260/230 in UV spectrum analysis usually suggests contamination in purified DNA. Thus, we believe that the purification protocol could benefit from specific optimization.

To check the quality of these purified tobacco leaf DNAs, we used qPCR to determine whether the amplification efficiencies were the same for DNA purified using different columns and buffers. As shown in Fig 3C, amplification of the same amount of DNA purified using the Qiagen kit and using a filter paper recharged spin column with homemade buffers produced plots with nearly identical Cq value across the threshold (22.80 ± 0.02 and 22.78 ± 0.04, respectively). Based on the assumption that PCR amplicons double their amount after each PCR cycle, there is only 2% (± 5%) difference between PCR amplification efficiencies for these DNA samples. It was therefore determined that tobacco DNA purified using the filter paper-based spin column with homemade buffers contained no additional inhibitors for PCR as DNA purified using a standard commercial kit. DNA purified using recharged and homemade spin columns are more suitable for low throughput experiments, such as analysis of transgenic plants (S2 Fig).

In addition to genomic DNA purification, we also tested an in-house protocol and buffers described by Yaffe et al [14] for plant RNA purification. Purification of tobacco total RNA using a filter paper-based spin column with homemade buffers is similar, or superior, to the performance of the Qiagen Plant RNeasy mini kit (Fig 3D, Table 1). For instance, RNAs purified by filter paper recharged spin columns with homemade buffer exhibit the same rRNA banding patterns as that for samples purified by the Qiagen kit, indicating similar integrity. Methods of RNA purification using filter paper-based spin column with homemade buffers also produce slightly high final RNA yields per unit of starting sample (Fig 3D, Table 1).

Analysis of UV spectrums illustrate standard RNA absorbance curves for tobacco leaf RNA purified using filter paper recharged spin column with the Qiagen kit buffers or homemade buffers (Fig 3E). Also, 260/280 and 260/230 absorbance ratios are all over 2.0 for these RNAs, which is indicative of high quality of RNA (Table 1).

The qRT-PCR analysis indicated no significant difference in amplifications between RNA purified by the Qiagen kit and the RNA purified using a filter paper-based spin column with homemade buffers. The Cq value of the reactions using the same amount of starting RNAs from these methods was similar (28.29 ± 0.09 and 28.61 ± 0.13) with the similar cross point of their amplification curves with a threshold (Fig 3F). There was a slight reduction at the level of 19% (± 12%) in the combined efficiency of reverse transcription and qPCR for RNAs purified using filter paper-based spin column with homemade buffer compared to RNAs purified using a Qiagen kit, however.

These results confirmed that filter paper-based spin columns can be adopted for purification of plant RNAs following a commercial kit protocol using kit buffers or an in-house protocol using homemade buffers. RNA purified by filter paper-based spin columns using homemade buffers is be suitable for many downstream experiments, including RT-PCR analysis of transgene expression in a transgenic plant, RACE for cDNA cloning, and even for construction of mRNA deep sequencing libraries (S3 Fig).

## Discussion

Development of filter paper-based spin columns is useful for nucleic acid purification because spin columns are widely used and associated methodology aligns with conventional desktop centrifuge equipment for low throughput bench-scale experiments involving nucleic acids. Recharged or homemade filter paper-based spin columns can be integrated with commercial kits or with in-house protocols, replacing expensive commercial spin columns that are typically repurchased after each use. The current price per commercial spin column is approximately $1 on the U.S. market. In comparison, filter paper discs cost less than 1 cent per column, a cost that is approximately 10 times less than glass fiber filters. Plastic ware of homemade spin column is costs approximately $0.2 per unit. All plastic ware associated with homemade spin columns can be easily disassembled, cleaned with bleach solution, and reused with little extra cost, however. This reduces the total cost for recharged or homemade filter paper-based spin column to under $0.1 per unit. Additional labor in spin column preparation should be considered manageable for low throughput applications, especially following the simplified recharging and assembly processes without adding a plastic fixing ring and support frit, which are required to fix and support soft silica-based binding materials (e.g. glass fiber filter) in commercial products.

With the availability of recharged or homemade filter paper-based spin columns, researchers can make cost-efficient use of laboratory resources by taking advantage of the extra buffer from commercial kits without additional cost, or by using less costly homemade buffers. Notably, homemade spin columns can be used with reduced sample amounts and half the normal buffer volumes. Homemade spin columns can yield nucleic acids at μg scale which is sufficient for many downstream applications.

By using recharged or homemade filter paper-based spin column along with leftover of commercial kit or homemade buffer solutions, the total expense for purification can be as low as $0.7 per DNA sample (with RNase as the major contributor to the final cost), and less than $0.5 per RNA sample. In contrast, typical commercial kits based on spin columns, such as the Qiagen kit, currently require approximately $4 per DNA sample and $7 per RNA in the United States. Some laboratories may be faced with greater costs, or have limited access to such kits, especially those in developing countries.

As a solid phase nucleic acid purification method, filter paper-based spin column eliminates the use of toxic solvents, such as chloroform or phenol, and thus is safe to conduct. Punching filter paper discs is a safe process. Punching and handling silica-based glass fiber filters can release airborne glass fibers, which is a potential hazard [22]. Also, recycling used spin columns reduces laboratory plastic waste.

Filter paper has weak binding ability to nucleic acids in lysates without adding binding buffers such as those used in the protocols of Qiagen DNeasy or RNeasy plant kits. Therefore, we used filter paper spin columns to filter cell debris instead of using QIAshredder spin column from the Qiagen kits. This additional filtering step might reduce final yield by about 10% based on experiments using cleared lysates, but it could help to increase the amount of cleared lysate for samples which are hard to clean by centrifugation only.

Some issues require caution in the application of the filter paper recharged or homemade spin columns. The first issue is centrifuge speed for recharged spin column with conical/V-shape bottoms or homemade spin columns based on 0.5 ml tubes. Such columns should only be centrifuged at relatively lower speed when filled with solution. We find it appropriate to centrifuge these columns at speeds not greater than 8000 × g. Otherwise, the solution may leak without passing through the filter and reduce the final yield of nucleic acids. However, when centrifuge to dry spin columns, we find it acceptable to centrifuge at full speed (> 16000 × g). Spin columns with net structures at the bottom to support filter discs (Fig 1A) can also be centrifuged at the full microcentrifuge speeds. However, in steps for binding plant genomic DNA or RNA, we still prefer relative lower speeds (not more than 8000 × g) during the binding and washing steps, except for the last drying filter paper step, which requires full speed to eliminate residual ethanol.

Purified DNA can sometimes contain RNA which can be observed as a small molecule smear in agarose gel electrophoresis and that can cause artificially inflated UV absorbance ratio at 280/260 nm. Although DNA containing RNA does not typically affect PCR, it may lead to an overestimate of DNA concentration. In such cases, we recommend reducing the amount of sample, such as using 50 mg per purification experiment or extending the time of cell lysis. Adding more RNase is not economical because the RNase is the major expense in purification using a homemade buffer. For instance, 4 μl RNase from Qiagen currently costs more than 30 cents. Purified DNA can also be quantified in other ways for more accurate concentration estimates for experiments sensitive to DNA input. For example, with tobacco DNA, we can use a fluorometer and an H33258 dye-based assay to quantify tobacco leaf genomic DNA for PCR based molecular marker analysis [23]. DNA quantification based on UV absorbance at 280 nm might not be consistent for DNA purified from different plant species using different commercial kits [24].

DNA contamination needs to be considered as a potential problem for RNA purification [25]. Although we found that filter paper-based spin columns do accommodate an in-column DNA digestion protocol as suggested in the Qiagen kit manual, we recommend treating RNA elution using a DNA-free kit of Invitrogen or similar kits to eliminate remaining DNA. This step is important for DNA contamination sensitive experiments, such as qRT-PCR for quantifying low expressed genes. If the abundance of a detected transcript is much greater than the background DNA [13, 21], the DNase treatment might be ignored in favor of using a negative reverse transcription control, or RT-PCR analysis using one gene-specific primer with one arbitrary primer annealing adaptor sequence added to mRNAs, such as Rapid Amplification of cDNA ends (RACE).

Nucleic acid purification using filter paper is mainly associated with cellulose, which appears to share similar features as silica-based materials in the binding of nucleic acids in chaotropic condition. However, only secondary fibril-associated cellulose within filter paper was found to isolate nucleic acids [5]. We found filter paper to be more efficient in the purification of high molecular weight plant genomic DNA, which is consistent with the report from Promega's Paramagnetic cellulose, which improves DNA yield for plant species [6]. Also, we found that filter paper is less efficient for plasmid DNA purification following a miniprep protocol. Reasons for this observation are unclear, however. These results suggest that cellulose-based material may bear some differences in mechanisms as compared silica-based material in binding and elution of nucleic acids. Unlike the silica-based nucleic acids purification process that has been extensively studied [26], there are few reports investigating the interaction between cellulose and nucleic acids [5, 13]. Therefore, more studies are needed to facilitate application of filter paper-based nucleic acid purification.

## Conclusions

We found filter paper to be an appropriate substitute for silica-based materials for purification of nucleic acids from various sources. Filter paper can be easily adopted to recharge used spin columns or to prepare homemade spin columns for low throughput applications using commercial kit buffers or homemade buffers to reduce laboratory costs. Filter paper can therefore be an important component for nucleic acid purification in molecular biology laboratories.

## Supporting information

S1 Fig Illustration of assembling different formats of filter paper-based spin column. (A) Recharged spin column with a flat bottom and net structure to support filter paper discs with diameter of 5/16 inch (~8mm) at position indicated with “*” label. (B) Recharged spin column with a V-shaped bottom using paper discs with diameter of 5/16 inch. (C) Recharged microspin column using filter paper with diameter of 5/16 inch or 3/16 inch at position indicated with “*” label. (D) Prepare homemade filter paper-based spin column prepared using 0.5 ml tube. (E) 10 μl TipOne pipette tip (Cat 1160–3700, USA Scientific) and head part of tip used as supporting ring to support filter paper discs in homemade filter paper-based spin column.
(TIF)

S2 Fig PCR Analysis of transgenic tobacco DNAs purified using filter paper based homemade spin columns and homemade buffer. Lane from left to right is PCR product of GUS fragment from pBI121 binary vector plasmid (positive control), and then PCR product from wild type tobacco DNA (negative control), followed lanes are PCR products of GUS amplified from DNAs of putative transgenic tobacco plant purified using filter paper based homemade spin column with homemade buffer. The right lane is 100 bp DNA marker (two strong bands are 1kb and 500bp in size).
(TIF)

S3 Fig Tapestation QC results for RNAseq libraries. **(**A) Gel image, (B) peak plot and (C) region table of RNAseq library prepared using RNA sample purified by Qiagen kit. (D) Gel image, (E) peak plot and (F) region table of RNAseq library prepared using RNA sample purified using recharged filter paper spin column with homemade buffer. These RNAseq libraries were constructed by Genomic Sciences Laboratory of North Carolina State University using NEBNext Ultra Directional RNA library Prep Kit for Illumina (New England BioLabs) followed the protocol for long insertion size option. Quality of RNAseq libraries was evaluated using Agilent 2200 TapeSation.
(TIF)
