# Supplementary figures and images for: Filter paper-based spin column method for cost-efficient DNA or RNA purification
Source: PLoS One. 2018 Dec 7;13(12):e0203011. doi: 10.1371/journal.pone.0203011 (PMC6286138; doi:10.1371/journal.pone.0203011)

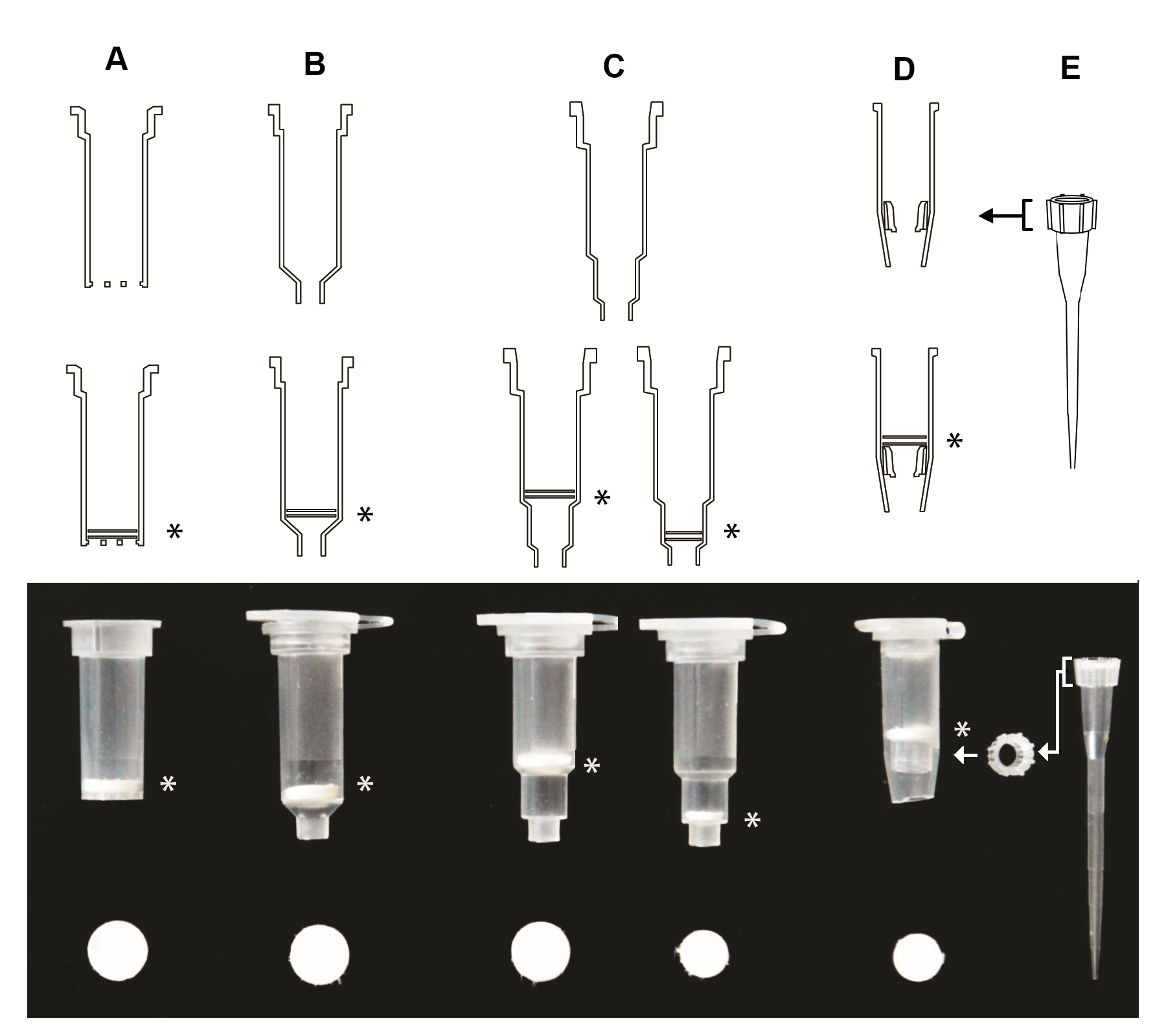

Supplement: S1 Fig — (A) Recharged spin column with a flat bottom and net structure to support filter paper discs with diameter of 5/16 inch (~8mm) at position indicated with “*” label. (B) Recharged spin column with a V-shaped bottom using paper discs with diameter of 5/16 inch. (C) Recharged microspin column using filter paper with diameter of 5/16 inch or 3/16 inch at position indicated with “*” label. (D) Prepare homemade filter paper-based spin column prepared using 0.5 ml tube. (E) 10 μl TipOne pipette tip (Cat 1160–3700, USA Scientific) and head part of tip used as supporting ring to support filter paper discs in homemade filter paper-based spin column. (TIF) [file pone.0203011.s001.tif]

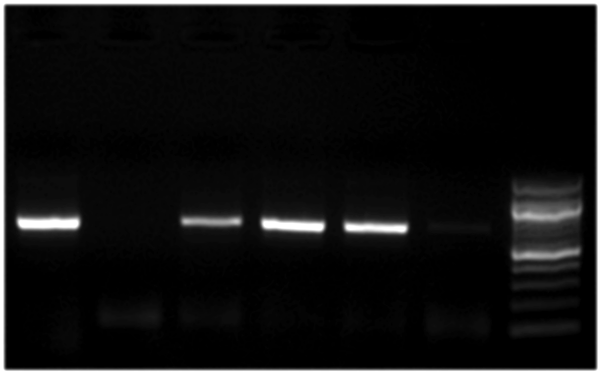

Supplement: S2 Fig — Lane from left to right is PCR product of GUS fragment from pBI121 binary vector plasmid (positive control), and then PCR product from wild type tobacco DNA (negative control), followed lanes are PCR products of GUS amplified from DNAs of putative transgenic tobacco plant purified using filter paper based homemade spin column with homemade buffer. The right lane is 100 bp DNA marker (two strong bands are 1kb and 500bp in size). (TIF) [file pone.0203011.s002.tif]

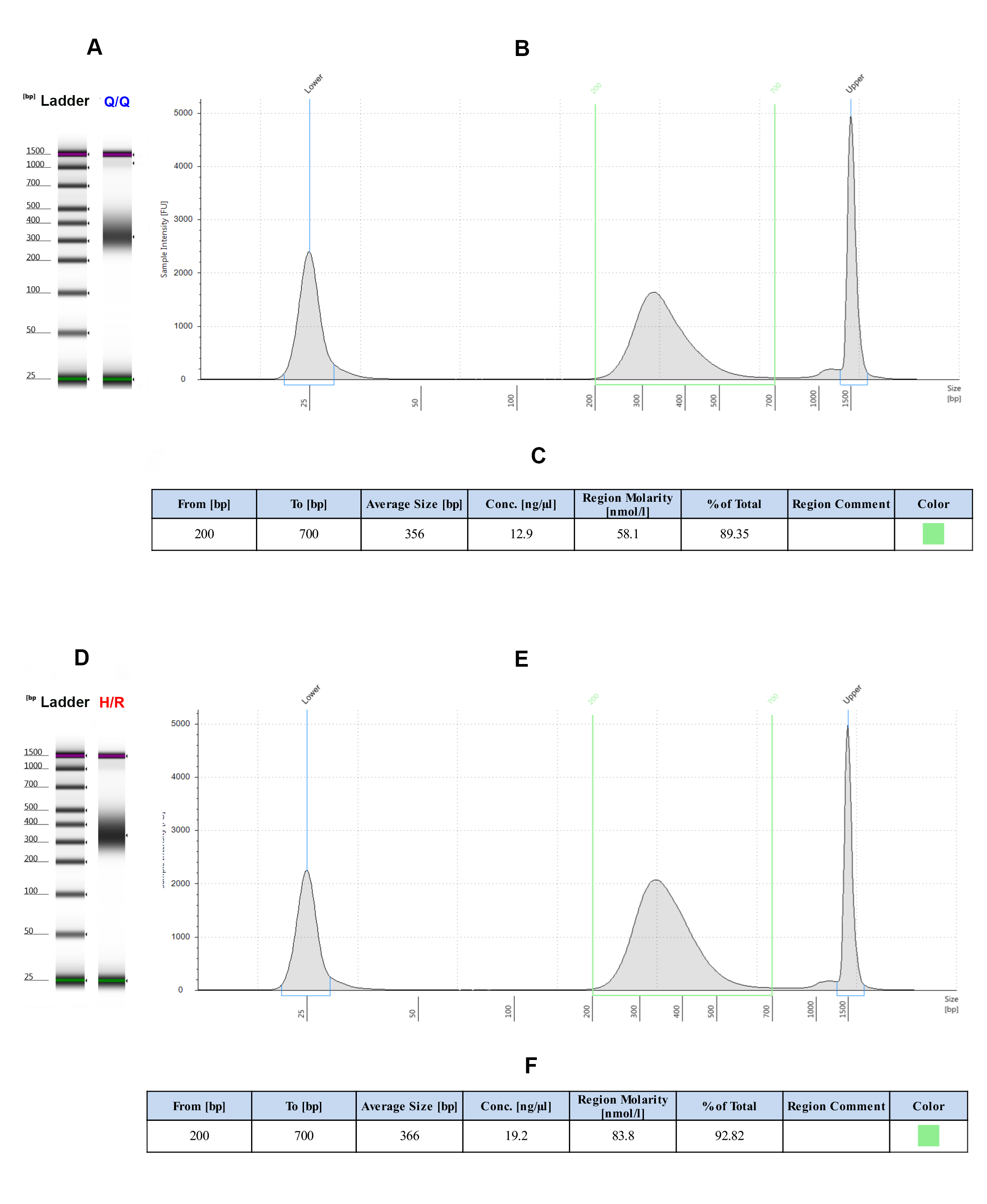

Supplement: S3 Fig — (A) Gel image, (B) peak plot and (C) region table of RNAseq library prepared using RNA sample purified by Qiagen kit. (D) Gel image, (E) peak plot and (F) region table of RNAseq library prepared using RNA sample purified using recharged filter paper spin column with homemade buffer. These RNAseq libraries were constructed by Genomic Sciences Laboratory of North Carolina State University using NEBNext Ultra Directional RNA library Prep Kit for Illumina (New England BioLabs) followed the protocol for long insertion size option. Quality of RNAseq libraries was evaluated using Agilent 2200 TapeSation. (TIF) [file pone.0203011.s003.tif]
